# Intestinal Microbiome Associated With Immune-Related Adverse Events for Patients Treated With Anti-PD-1 Inhibitors, a Real-World Study

**DOI:** 10.3389/fimmu.2021.756872

**Published:** 2021-12-16

**Authors:** Wenhui Liu, Fang Ma, Bao Sun, Yiping Liu, Haoneng Tang, Jianquan Luo, Huiqing Chen, Zhiying Luo

**Affiliations:** ^1^ Department of Pharmacy, The Second Xiangya Hospital, Central South University, Changsha, China; ^2^ Institute of Clinical Pharmacy, Central South University, Changsha, China; ^3^ Department of Oncology, The Second Xiangya Hospital, Central South University, Changsha, China; ^4^ Department of Laboratory Medicine, The Second Xiangya Hospital, Central South University, Changsha, China

**Keywords:** gut microbiome, PD-1, PD-L1, immune-related adverse effects, interindividual difference

## Abstract

**Aim:**

Immune checkpoint inhibitors (ICIs) have updated the treatment landscape for patients with advanced malignancies, while their clinical prospect was hindered by severe immune-related adverse events (irAEs). The aim of this study was to research the association between gut microbiome diversity and the occurrence of ICI-induced irAEs.

**Patients and Method:**

We prospectively obtained the baseline fecal samples and clinical data from patients treated with anti-PD-1 inhibitors as monotherapy or in combination with chemotherapy or antiangiogenesis regardless of treatment lines. The 16S rRNA V3-V4 sequencing was used to test the gene amplicons of fecal samples. The development of irAEs was evaluated and monitored from the beginning of therapy based on CTCAE V5.01.

**Results:**

A total of 150 patients were included in the study and followed up for at least 6 months. A total of 90 (60%) patients developed at least one type of adverse effect, among which mild irAEs (grades 1–2) occurred in 65 patients (72.22%) and severe irAEs (grades 3–5) in 25 patients (27.78%). Patients with severe irAEs showed a visible higher abundance of *Streptococcus*, *Paecalibacterium*, and *Stenotrophomonas*, and patients with mild irAEs had a higher abundance of *Faecalibacterium* and unidentified_*Lachnospiraceae*. With the aid of a classification model constructed with 5 microbial biomarkers, patients without irAEs were successfully distinguished from those with severe irAEs (AUC value was 0.66).

**Conclusion:**

Certain intestinal bacteria can effectively distinguish patients without irAEs from patients with severe irAEs and provide evidence of gut microbiota as an informative source for developing predictive biomarkers to predict the occurrence of irAEs.

## Introduction

In recent decades, immunosuppressive therapy has dramatically prompted a paradigm shift in therapy of cancer diseases (1, 2). Immune checkpoint inhibitors (ICIs), including antiprogrammed death 1 (anti-PD-1), antiprogrammed death 1 ligand (anti-PD-L1) and cytotoxic T-lymphocyte-associated protein 4 (CTLA-4) inhibitors, have been approved as first-line treatment strategy for a variety of advanced cancers, such as melanoma, gastric cancer, and hematological malignancies (3). ICIs play antitumor effect through boosting the body’s natural defense against carcinoma cells. The high-speed development of immune checkpoint therapies was owing to their inspiring clinical efficacy in numbers of tumors (4, 5). It has been widely evidenced that several host factors, including the expression level of PD-L1, tumor mutational burden (TMB), microsatellite instability (MSI), and gut microbiome diversity, can be used to predict the treatment outcomes of ICI treatment (6, 7).

As only part of patients can benefit from ICI monotherapy, ICIs plus chemotherapy, or antiangiogenesis has been approved as successful first-line therapy for several malignant tumors regardless of the expression level of PD-L1 in tumor tissues (8–10). However, the treatment benefits associated with ICIs come at the cost of immune-related toxicities (known as irAEs), which are distinctly different from chemotherapy-related toxicities, and been regarded as the off-target effects of an excessively activated immune system (11). The increased efficacy of combination therapy is accompanied by a rising incidence of irAEs, especially severe or life-threatening irAEs (8, 12, 13).

Considering that serious irAEs are one of the main reasons for patients withdrawing treatment or death, the detailed molecular mechanism underlying the selectivity of irAEs during anti-PD-1/PD-L1 treatment is urgently needed (14). Numerous studies have evidenced that irAEs had good prognostic value in different cancers and could be used as valuable biomarkers in clinical setting (15–17). Several factors associated with the antitumor efficiency of ICIs, including TMB, the neutrophil-to-lymphocyte ratio (NLR) and platelet-tolymphocyte ratio (PLR) levels, have also been evidenced as independent risk factors related to the occurrence of irAEs (18, 19). Moreover, a large number of preclinical and clinical studies evidenced that the antitumor effects of ICIs depended on gut microbiome *via* innate and adaptive immunity, and microbiome modulation by microbiota transplantation experiments could improve therapeutic responses of ICIs (20–22). Moreover, a recent study found that in patients with advanced nonsmall-cell lung cancer (NSCLC) and treated with anti-PD-1/PD-L1 antibodies as a first-line or treatment-refractory therapy, and there is a difference in gut microbiome between patients having low- and high-grade irAEs (23). Collectively, we speculate that the mechanism of irAEs may partly be consistent with the pharmacology effects of these drugs depending on the gut microbiome.

Currently, a total of 8 PD-1/PD-L1 inhibitors have been used in China for antitumor treatment (6 were anti-PD-1 and 2 were PD-L1 inhibitors), and anti-PD-l was one of the most commonly used ICIs in China. This study intended to analyze the gut microbiome data from patients undergoing anti-PD-1 inhibitor therapy who suffered from irAEs and those who did not and to explore the possible relationship between gut microbiota and the occurrence of irAEs. Moreover, we also aim to point out the potential difference in microbiota composition among various types of irAEs. On this basis, the study will attempt to identify suitable microbial biomarkers from gut microbiota to construct a severe irAE classification model in addition to developing a simple and noninvasive technique to predict severe irAEs before taking anti-PD-1 inhibitors.

## Method

### Study Population

A unicentric and prospective observational study was conducted to elucidate the effect of gut microbiota on severe irAE interindividual differences in the Chinese population. We collected the fecal samples from patients who acquired anti-PD-1 inhibitor (nivolumab or pembrolizumab) therapies between October 2018 and March 2021 in the Department of Oncology, Second Xiangya Hospital, Central South University (Changsha, China). All of them received anti-PD-1 as monotherapy or in combination with chemotherapy or antiangiogenesis (bevacizumab or anlotinib) regardless of treatment lines. The anti-PD-1 was administered intravenously every 3 weeks at a dose of 5 or 10 mg/kg. This study was approved by the Ethics Committee of Second Xiangya Hospital at Central South University, and all procedures were carried out under the Declaration of Helsinki. The study was registered with the Chinese Clinical Trials Registry (ChiCTR2100045873).

All participating patients were given informed consent to this study. Patients were included according to the following criteria: (1) clinical symptoms, physical signs, imaging examination, and histologically or cytologically consistent with the diagnostic criteria for tumors; (2) treatment with anti-PD-1 inhibitor monotherapy or combination therapy at recommended dose; (3) no use of antibiotics or microbial ecological agents for at least 4 weeks before anti-PD-1 therapy; (4) no serious autoimmune diseases; (5) normal routine examination result of the stool before anti-PD-1 therapy.

### Patient Follow-Up and Definition of irAEs

The detailed demographics, medical history, and comorbidities were further collected by a review of electronic medical records, including age, sexual, smoking and drinking history, primary tumor sites, histological types, Eastern Cooperative Oncology Group (ECOG) performance status (PS), the expression level of PD-1, treatment strategies, comorbidities, and irAEs data of the last follow-up. The use of antibiotics, especially broad-spectrum antibiotics was also recorded. The follow-up lasted for at least 6 months by regular clinic visits conducted by an oncologist (Dr. Fang Ma) and a pharmacist (Pharm. Wenhui Liu) regularly.

The occurrence of irAEs was trailed and monitored from therapy start and only patients whose onset was before January 2021 were included in the irAEs group in this study. Cases with irAEs were reviewed by at least two oncologists and one clinical pharmacist specializing in antitumor. Chemotherapy-associated adverse effects, including hepatotoxicity, nephrotoxicity, hematotoxicity, and so on, were excluded for patients taking combination treatment. Only irAEs certainly or probably related to anti-PD-1 therapy were recorded, and their severity was assessed according to the National Cancer Institute Common Terminology Criteria for Adverse Events (CTCAE V5.0). IrAEs for patients taking anti-PD-1 inhibitor plus chemotherapy were evaluated by two oncologists independently and categorized by CTCAE V5.01. Severe irAEs (grades 3–4) were further confirmed if they could be cured by immunosuppression with corticosteroids or other immunosuppressant agents such as infliximab (24).

### Fecal DNA Extraction and 16S Sequencing

Using the commercial sampling kit containing guanidine solution, we prospectively collected fecal samples before patients had anti-PD-1 inhibitors therapy and stored the samples at −80°C until analysis. Bacterial DNA was extracted at Novogene Bioinformatics Technology Co., Ltd. (Cambridge, UK) using TIANGEN kit (catalog number: DP328) according to the manufacturer’s recommendations. The quality of isolated DNA was confirmed by agarose gel electrophoresis. DNA library was prepared using the PrepX ILM 32i DNA library kit (Wangfergen Biosystems, Fremont, CA, USA). V3 and V4 regions of 16S ribosomal RNA (rRNA) gene were amplified using the following primers: MI-16S-F (TACGGRAGGCAGCAG) and MI-16S-R (AGGGTATCTAATCCT). Sequencing was performed on the Illumina MiSeq platform (Illumina, San Diego, CA, USA).

16S sequencing data were analyzed using Software Quantitative Insights into Microbial Ecology. Operational taxonomic unit (OTU) counts per sample were generated and grouped by different taxonomic levels (phylum, class, order, family, genus, and species). The diversity of gut microbiome was assessed by alpha diversity and beta diversity. Indexes including Shannon and Inverse Simpson were calculated based on OTU counts. The difference in alpha diversity between groups was statistically analyzed by the Mann-Whitney U test (p < 0.05). Principal coordinate analysis (PCoA) plots using unweighted UniFrac distance were created to visualize the variation between different groups. Linear discriminant analysis effect size (LEfSe) analysis was used to identify the characteristic genera in different groups, and a score of log linear discriminant analysis (LDA) >2.0 or an odds ratio with p < 0.05 was considered to indicate a differential signature that was better discriminated between groups. The support vector machine (SVM) algorithm and receiver operator characteristic (ROC) curve calculation were performed by the RandomForest and ROCR packages in R (version 3.2.1), respectively, based on the species abundance in gut microbiome. The recursive feature elimination method was used to rank the importance of all bacteria and to draw the ROC curve. Finally, five-fold cross-validation with 1,000 iterations was used to evaluate the performance of these models.

### Statistical Analyses

The clinical results were expressed as mean and standard deviation for continuous variables and frequency and percentage for categorical variables. The *t*-test (for continuous variables) and *χ* ^2^ test (for categorical variables) were used to analyze the characteristic clinical difference between different groups. The R statistical Language (version 3.2.1) and GraphPad Prism (v6.0e) software packages were used to analyze the difference of intestinal microbiome profiling between groups. A nonparametric test was applied to determine the differences in the relative abundance of OTU counts and alpha diversity indexes between groups. The cutoff for the Shannon index was calculated based on receiver operating characteristic curves. The microbiota diversity was estimated by the Shannon index. Differences were considered to be statistically significant when *p* < 0.05.

## Results

### Clinical Characteristics of the Enrolled Patients

A total of 150 patients were included in the study and followed-up for at least 6 months. Patients in this study ranged in age between 17 and 79 years, and mean age was 57.53 ± 10.0 years. The cohort was predominantly male (85.33%). Most patients (57.3%) had a previous smoking history, and part of the patients (28.7%) had a prior drinking history. A total of 102 patients had (68%) been diagnosed with NSCLC; in addition, 7 (6.86%) had nasopharyngeal carcinoma, 5 (4.9%) had melanoma, 5 (4.9%) had esophagus cancer, and 31 (20.67%) with other cancer types. The majority of patients (92.67%) were diagnosed with advanced stage (stages III to IV) tumors, with patients (97.33%) having an ECOG PS of ≥1.

Seventy-eight (52%) patients had received at least one round of chemotherapy or radiotherapy before anti-PD-1 treatment was taken. A total of 132 (88%) patients were treated with anti-PD-1 (nivolumab or pembrolizumab) plus chemotherapy (mainly platinum-based chemotherapy, 73.48%), and 18 (12%) of patients received anti-PD-1 monotherapy. In total, 90 patients (60%) developed one or more irAEs; 72 (48%) patients stopped taking anti-PD-1 inhibitor therapy because of disease progression (62.5%), adverse events (29.2%) or financial burden (8.3%). The clinical characteristics of enrolled patients are summarized in **Table 1**.

**Table 1:** Characteristics of enrolled patients.

Characteristics	Patient count (N = 150), N (%)
Age	
mean±SD	57.53±10.0
Sex	
Male	128 (85.33%)
Female	22 (14.67%)
BMI, mean±SD	22.12±3.92
Smoke habit	85 (56.67%)
Drink habit	44(29.33%)
Disease stage	
I-II	11 (7.33%)
III-IV	139 (92.67%)
Cancer type	
Non-small-cell lung cancer	102 (68%)
squamous carcinoma	54 (52.94%)
adenomatous carcinoma	48 (47.06%)
Nasopharyngeal carcinoma	7 (6.86%)
Malignant melanoma	5 (4.9%)
Esophagus cancer	5 (4.9%)
Other types	31 (20.67%)
ECOG PS score before treatment	
0	4 (2.67%)
1	129 (86%)
≥2	17 (11.33%)
Patients with PD-L1 expression level	91 (60.67%)
<1%	33 (36.26%)
1%-49%	29 (31.87%)
≥50%	29 (31.87%)
Treatment naïve patients	73 (48.67%)
Therapeutic regimen	
anti-PD-1plus chemotherapy	128 (85.33%)
Platinum based chemotherapy	97 (75.78%)
Taxol	11(8.58%)
Anti-angiogenic	4 (3.12%)
Others	16 (12.5)
anti-PD-1 monotherapy	22 (14.67%)
irAEs	90 (60%)
irAEs (grade 3-4)	25 (27.78%)
irAEs (grade 1-2)	65 (72.22%)
Non-irAEs	60 (40%)
Drug discontinuance	72 (48%)
Because of AEs	14 (29.2%)
Because of disease progression	45 (62.5%)
Because of financial reasons	13 (8.3%)

BMI, body mass index; ECOG PS score, Eastern Cooperative, Oncology Group performance status; AE, adverse effect.

### IrAE Development in Patients Followed Up

**Figure 1:**
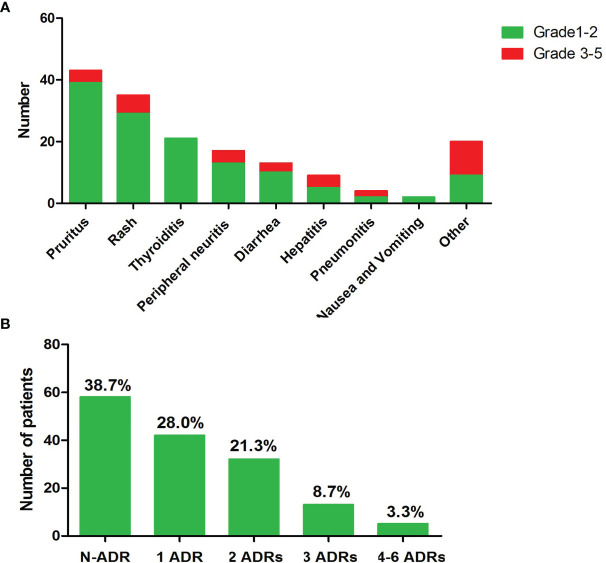
Overview of the occurred irAEs during follow-up. **(A)** Numbers of patients of each type irAE. **(B)** Numbers of occurred irAEs during follow-up time.

The median follow-up for this cohort was 8.4 months [interquartile range, 7.2–9.5 months]. The most common irAEs associated with anti-PD-1 inhibitor use are pruritus and/or rash, and thyroid dysfunction, as shown in **Figure 1A**. Ninety (60%) patients developed at least one type of adverse effect, among which 68 (72.22%) patients had mild irAEs (grades 1–2) and only 25 (27.78%) patients suffered from severe irAE (grades 3–5). Among them, most patients had one to two types of irAEs and only 5 patients suffered 4 to 6 kinds of irAEs, as shown in **Figure 1B**.

### Specific Gut Microbial Signature in Patients With irAEs

**Figure 2:**
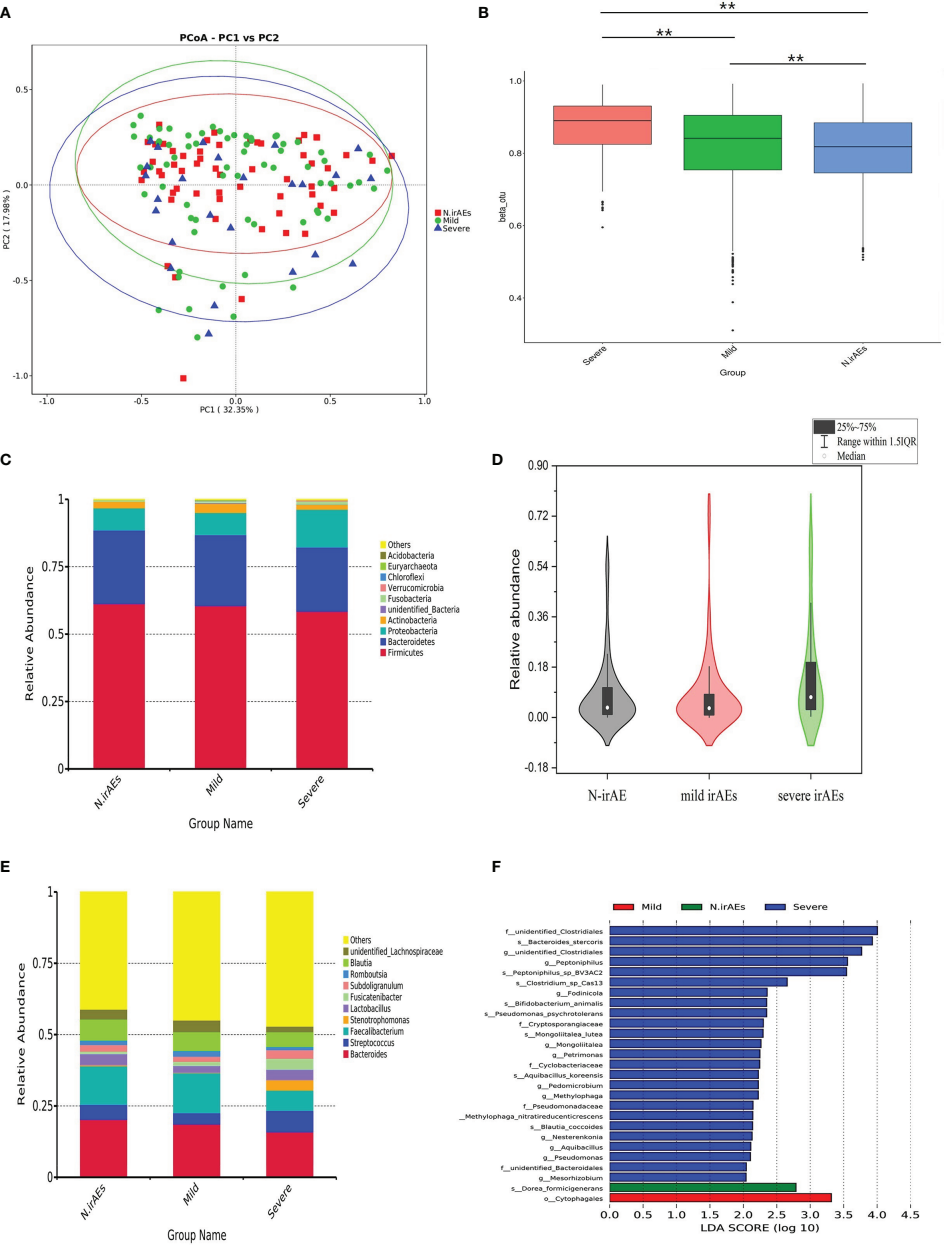
Gut microbiome composition for all patients stratified by irAEs. **(A)** PCoA test was used to measure the shift in intestinal bacterial composition profile among groups. **(B)** Bacterial community dissimilarities among groups. Bray-Curtis distances were independently calculated for N-irAEs vs. mild irAEs. Statistical significance was determined by the Mann-Whitney *U* test. ^**^ *p* < 0.001. **(C)** Phylogenetic composition of the top 10 bacterial taxa at the phylum level, ordered by the most abundance taxa across the cohort. **(D)** Relative abundance of *Proteobacteria* in the phylum level. Statistical differences were assessed by Wilcoxon test. **(E)** Phylogenetic composition of common bacterial taxa at the genus level, ordered by the most abundance taxa across the cohort. **(F)** Differential abundance analysis using LEfSe stratified according to the occurrence of irAEs. Note that all findings reported on LEfSe are statistically significant. LDA, linear discriminant analysis.

Patients have been divided into three groups, as shown in **Table 2**, and the clinical backgrounds were roughly similar among groups (**Table 2**). A total of 28 patients received antibiotics therapy during follow-up, while the ratio of antibiotics usage had no statistical discrepancy between groups. The rarefaction curves of different groups showed that the number of sequences could represent the microbial diversity of each community (**Supplementary Figure S1**). PCoA test showed that patients were divided into different clusters (**Figure 2A**), although there was no significant difference in α-diversity among groups (**Supplementary Figure S2**). The Bryan-Curtis intragroup distance of the no irAE (N-irAE) group was smaller than both the mild irAEs and severe irAEs groups (*p* < 0.001, by Mann-Whitney *U* test, **Figure 2B**). These results suggest that patients without irAEs have a distinctly different gut microbial community from those with mild and severe irAEs.

**Table 2:** Univariate analysis for irAEs in patients with different clinical factors.

Variable	N-irAEs group(N = 58)	Mild irAEs group (N = 67)	Sever irAEs group (N = 25)	*P*1	*P*2	*P*3
Age, year	57.71±9.56	57.18±10.34	58.16±10.23	0.91	0.79	0.84
BMI,	22.27±3.22	22.70±2.78	22.13±2.77	0.60	0.41	0.86
Sexual (man / female)	49/9	60/7	20/5	0.46	0.35	0.75
Smoking habit (yes / no)	32/26	42/26	10/15	0.17	0.12	0.24
Drink habit (yes / no)	17/41	20/47	6/19	0.85	0.64	0.79
Cancer type (Lung cancer)	34	50	19	0.11	0.48	0.14
Disease stage (I-III / IV)	26/32	29/28	9/16	0.28	0.19	0.48
PD-L1 level						
<1%	13	9	10			
1%-49%	8	15	6			
≥50%	15	14	0			
Treatment strategy (yes / no)						
Treatment naïve	31/27	29/36	13/12	0.59	1.0	1.0
Monotherapy	7/51	13/54	2/23	0.29	0.53	0.72
ICIs plus platinum based chemotherapy	37/21	42/25	18/7	0.70	0.18	0.61
ICIs plus taxol (yes / no)	2/56	6/61	3/22	0.29	0.39	0.16
Antibiotics usage (yes / no)	7/51	13/54	8/17	0.10	0.088	0.058

N-irAEs group, patients without irAEs; mild irAEs group, patients occurred grade 1 or 2 levels irAEs; severe irAEs group, patients occurred grade 3 to 5 level irAEs; P1, N-irAEs vs. mild irAEs vs severe irAEs; P2, (N-irAEs plus mild irAEs) vs severe; P3, N-irAEs vs severe irAEs;treatment naïve, patients who didn’t take any anti-cancer treatment before treated with anti-PD-1 inhibitors.

### Difference in the Gut Microbiome Between Patients With irAEs

In detail, patients without irAEs or with mild irAEs showed a similar abundance of the top 10 abundant bacteria in the phylum level, which differed from patients with severe irAEs (**Figure 2C**). The abundance of *Proteobacteria*, for example, was higher in severe-irAEs group compared with N-irAEs or mild-irAEs groups (*p* = 0.032, Mann-Whitney *U* test, **Figure 2D**). Patients with severe irAEs showed a visible abundance of *Streptococcus*, *Paecalibacterium*, and *Stenotrophomonas* at the genus level, while patients with grades 0–2 irAEs had a higher abundance of *Faecalibacterium* and unidentified_ *Lachnospiraceae* at the genus level (**Figure 2E**). These results suggested that patients with severe irAEs had an intestinal microbial community significantly different from those without or with mild irAE.

To identify specific microbial biomarkers that can be used to classify patients with or without irAEs, the differential genera between groups were further investigated by LEfSe analysis with the threshold value of LDA 2.0. The abundance of bacteria taxa with significant difference among groups are shown in **Figure 2F**. A total of 22 differential markers had significantly different abundance between patients in N-irAEs and mild irAEs groups: 8 enriched in mild irAEs group including *Nocardiaceae* and *Pseudomonadaceae*, and 14 enriched in N-irAEs group including *Balneolales* (**Supplementary Figures S3A, B**). Furthermore, 40 differential markers showed significant different abundance between N-irAEs and severe irAEs groups, 35 enriched in severe irAEs group including *Spirosomaceae, Thermoanaerobacteracea*, Anaplasmataceae, and *Vibrionales* and 5 enriched in N-irAEs group including *Pseudomonadales* (**Supplementary Figures S3C, D**).

### Difference in the Gut Microbiome Between Patients With Each irAE Subtypes

Based on the diverse mechanism of each kind of irAEs, we next compared the microbial compositions in adverse effect subtypes (pruritus, rash, thyroid dysfunction, and diarrhea) separately. For pruritus, the observed species number of intestinal bacteria showed no significant difference between groups (**Supplementary Figure S4A**). Still unweighted UniFrac analysis revealed that a significant difference in intestinal gut bacteria composition between N-irAEs group and the pruritus group (*p* = 0.024, **Supplementary Figure S4B**). Both PCoA plot and the abundance of top 10 bacteria in the genus level did not have a noticeable difference between groups (**Supplementary Figures S4C, D**). LEfSe analysis indicated that a total of 31 differential bacteria markers showed a significant difference between N-irAEs and pruritus groups, with 16 markers enriched in the pruritus group and 6 enriched in N-irAEs group (**Supplementary Figure S4E**).

For rash, there is no significant difference in α-diversity and β-diversity between patients with rash and those without irAEs (**Supplementary Figures S5A–D**). LEfSe analysis showed a substantial difference between N-irAEs and rash groups in a total of 31 differential bacteria markers, and 26 enriched in patients with rash and 5 enriched in the N-irAEs group (**Supplementary Figure S5E**).

For thyroid dysfunction, the observed species numbers of intestinal bacteria had no significant difference between groups (**Supplementary Figure S6A**), while unweighted UniFrac analysis showed that intestinal gut bacteria composition was significantly different between groups (*p* = 1.98E−17, **Supplementary Figure S6B**). Further PCoA plots showed a visible separation of bacterial taxa composition between patients without irAEs and those with thyroid dysfunction (**Supplementary Figure S6C**). The abundance of top 10 bacterial in the genus level varied between groups (**Supplementary Figure S6D**): the N-irAEs group had a higher abundance of *Bacteroides* and *Lactobacillus*, while *Paecalibacterium* was enriched in the thyroid dysfunction group. LEfSe analysis identified 5 differential bacteria markers showing a significant difference between N-irAEs and thyroid dysfunction group: the g-*Ralstonia* and k-*Bacteria* were enriched in patients with thyroid dysfunction, and the N-irAEs group was rich in o_*Micrococcales*, g_*Granulicatella*, and f_*Carnobacteriaceae* (**Supplementary Figure S6E**).

For diarrhea, 10 patients were having mild diarrhea and 3 patients had severe diarrhea. In terms of the observed species number of intestinal bacteria, there was no significant difference among groups (**Supplementary Figure S7A**), while unweighted UniFrac analysis showed that intestinal gut bacteria composition were significantly different among groups (*p* < 0.001, **Supplementary Figure S7B**). Patients with severe diarrhea showed a higher level of *Stenotrophomonas* and *Streptococcus* compared with patients without irAEs or with mild diarrhea (**Supplementary Figure S7C**), while the abundance of *Faecalibacterium* and *Bacteroides* was higher in patients without irAEs or with mild diarrhea. Furthermore, LEfSe analysis showed a range of bacteria taxa showed obvious differences among groups (**Supplementary Figure S7D**).

### Classification Models of Severe and Mild irAEs Based on SVM Algorithm

**Figure 3:**
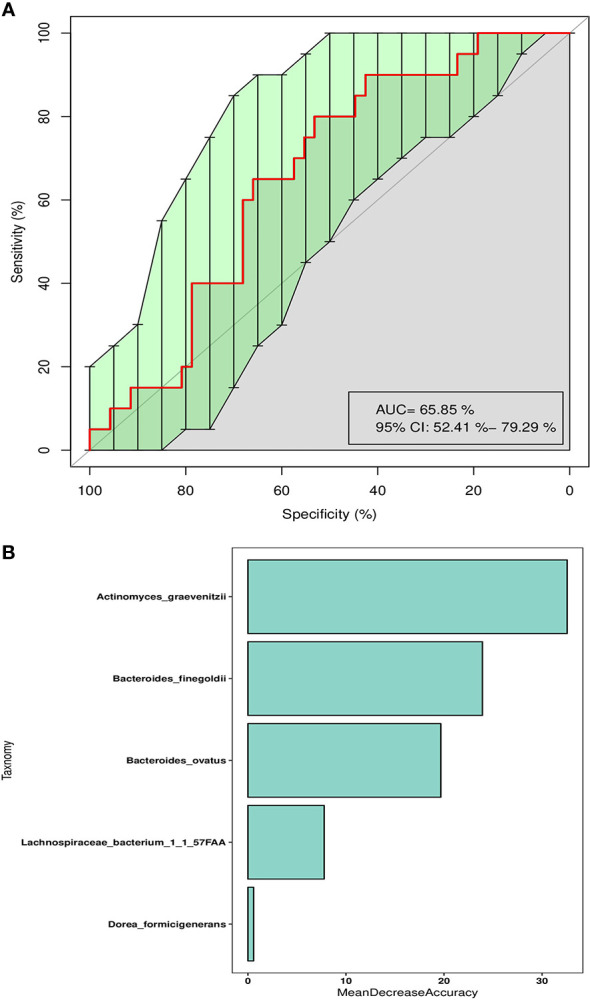
Classification model of severe irAEs based on intestinal microbes. **(A)** The ROC curve of SVM classification models using the species abundance in the train set. **(B)** The mean decrease accuracy of each enrolled bacteria taxa in the SVM classification model.

Collectively, previous results indicate that intestinal bacterial structure was associated with the occurrence of irAEs, especially severe irAEs. As many researchers emphasized that a crucial aspect in the future development of ICI therapies is to improve the understanding of events leading to irAEs, we further aimed to develop a classification model by machine learning method to evaluate the usability of gut microbiome as prognostic biomarkers for severe irAEs. To construct this model based on the SVM algorithm, we firstly find the optimal combination of microbial biomarkers to optimize the efficiency of severe irAEs identification. We performed SVM test based on different bacterial features at the species level, and found these five microbial biomarkers (*Actinomyces_graevenitzii*, *Dorea_formicigenerans*, *Bacteroides_ovatus*, *Bacteroides_finegoldii*, *Lachnospiraceae bacterium 1_1_57FAA*) had the best prediction ability (**Supplementary Figures S8A, B**). The AUC value of the combination of these bacterial features was 65.85% (95% CI: 52.4%–79.29%) in the train set (**Figure 3A**) and as high as 0.73 (95% CI: 0.43–1) in the test set (**Supplementary Figure S8C**). Of these five microbial biomarkers, the *Actinomyces_graevenitzii* and *Bacteroides_finegoldii* contribute most to the model (**Figure 3B**).

We constructed classification models to predict the microbial condition of patients with mild irAEs based on the same process. We found that a classification model with five microbial biomarkers had the best prediction ability (**Supplementary Figures S9A, B**). The AUC value of combining these bacterial features was as high as 66% (95% CI: 55.16%–76.83%), as shown in **Supplementary Figures S9C, D**.

## Discussion and Conclusion

In this prospective study, we reported a comprehensive analysis of the gut microbiomes of patients receiving anti-PD-L1-based treatment. We observed that the gut microbiome composition was significantly different among patients without irAEs or with mild/severe irAEs. In addition, we further found different gut microbiome compositions in various types of irAEs. Finally, we constructed a classification model with 5 microbial biomarkers, which could successfully distinguish patients without irAEs from those with severe irAEs.

The occurrence of irAEs is positively correlated with the clinical response of ICIs. Hence, we speculate that there is an intersection between the mechanisms of irAEs and treatment response. In accordance with our hypothesis, we found that the relative abundance of bacteria taxa showed significant difference among groups. We first observed that the relative abundance of *Faecalibacterium* genus tended to be more enriched in patients without irAEs or with mild irAEs. *Faecalibacterium* genus was a Gram-positive bacterium of the Ruminococcaceae family and within the Clostridia class of the Firmicutes phylum. *Faecalibacterium prausnitzii* (*F. prausnitzii*), the only known species of the *Faecalibacterium* genus, had been widely proved to be positively associated with good treatment response of ICIs and CD8+ T-cell infiltration within the tumor microenvironment (20, 25). The abundance of *F. prausnitzii* was likely to be associated with better treatment response and a lower risk of severe irAEs. However, there was also evidence that patients with metastatic melanoma who had a good response to ICIs had enrichment of this bacterium in baseline stool and higher incidence of immune-related colitis (26).

In our cohort, several bacteria taxa (*Streptococcus*, *Paecalibacterium*, and *Stenotrophomonas*) enriched in patients with severe irAEs were also reported in other anti-PD-L1-associated clinical studies of different cancer types. A metagenomics study, for example, found an elevated abundance of *Streptococcus* in melanoma patients who responded to ICIs (22). However, one recently published research with a small sample size showed a negative correlation between the abundance of *Streptococcus* genus and treatment response in patients with unresectable metastatic melanoma treated with ICIs (27). Numbers of factors including the study design, sample size, sample collection, treatment regimen, regional differences, and data analysis method might further complicate the interpretability of finding on microbiome compositions among different studies.

The frequency and predominance of irAEs vary among ICIs targeting CTLA-4 and anti-PD-1/PD-L1 (e.g., anti-CLTA-4 inhibitor is linked to a higher rate of colitis whereas anti-PD-1 has a higher rate of pneumonitis) (28). Higher abundance of *Bacteroidetes* in the phylum level was widely evidenced in patients who did not develop ICI colitis (29). Similarly, our research also showed that patients with grades 0–2 diarrhea showed significantly higher *Bacteroides* and *Faecalibacterium* than patients who developed severe diarrhea associated with anti-PD-1 treatment. Based on these, we can speculate a crossover in the pathogenesis between anti-CTLA4-induced colitis and anti-PD-1-induced diarrhea.

Exposure to antibiotic therapy adversely influenced outcomes of ICI therapy through modulation of intestinal microbiota. Numbers of clinical studies had indicated that prior antibiotic treatments were associated with worse treatment response and OS in patients treated with ICIs (30), as well as greater incidence of moderate to severe irAEs (31). As the timing of antibiotic exposure was crucial in determining degree of its impact on the ICIs response, we collected patients who did not receive antibiotics 4 weeks before anti-PD-L1 treatment in this study. Pinato et al. found that concurrently received antibiotics were not associated with response to ICI therapy or survival in patients with cancer. We failed to evidence the association between coadministration of antibiotics and the occurrence of irAEs, as the ratio of antibiotics usage showed no significant difference between groups. Further studies should be designed to explore the influence of concurrent antibiotic uses on the toxicity of ICIs.

An essential aspect of studying the gut microbiome and its relationship with the human host is to derive biomarkers for diagnostic or prognostic prediction. Differential bacteria taxa between groups can help identify relevant microbial species as potential biomarkers. It is very encouraging to see that our model achieves desirable performance in this cohort, indicating that the gut microbiome systematically affects host immune function, and it may serve as a repertoire for novel biomarkers of severe irAEs. The wider intervals indicate uncertainty of the predictive model in this study, which may be due to the high intersample variability. Another possible reason may be the lack of irAE-associated clinical features. Future enlargement of the study cohort is expected to improve the prediction accuracy further.

A previous study showed that polymorphisms in human leukocyte antigen (HLA) genes were associated with irAEs in patients under immune checkpoint therapy (32). There was also evidence that major histocompatibility complex (MHC) polymorphisms differentially influence antibody-mediated selection on the microbiota in mice model (33). Multiple mechanisms, including regulation of the cytotoxic activity, humoral responses and inflammatory reaction have been proposed to be involved in irAEs (34). However, little evidence has been disclosed about the mechanisms of irAEs, such aHs whether those immune cells mainly responsible for irAEs are also participating in the potentiating of the antitumor immune response. Recently, Alexandre and colleagues proposed an interesting and thought-provoking viewpoint that the symptoms of irAEs were very similar to a chronic graft-versus-host-disease (GVHD) reaction induced in the context of allogeneic bone marrow transplantation (35). Low-dose ICIs could induce a prolonged auto-GVHD, which would improve the antitumor efficacy of the patients’ own lymphocytes for a broad spectrum of malignancies (36, 37). It is challenging to explain the internal interaction of irAEs and the more complicated question is the different pathophysiological mechanisms under various types of irAEs.

Our study has several limitations. Firstly, we did not collect the fecal samples from patients at the time point when irAEs have occurred, then we could not correlate the change of gut microbiota composition with the disease course of irAEs. Secondly, we could not correlate the gut microbiota composition with patients’ dietary history. Neither did we find any clinical features associated with the occurrence of irAEs, which might be due to a low sample size of our study. We planned to research the effects of patients’ dietary habits and concomitant medication on the occurrence of irAEs in the next step. Thirdly, 16S rRNA sequencing might be underpowered to illustrate the whole gut microbiota signature. Metagenomics sequencing is likely to be a more profound gut microbiota profiling technique in finding more irAE-correlated biomarkers. Further studies are needed to find biomarkers or models that can predict patients with good therapeutical effects as well as reduce the risk of irAEs. A thorough understanding of irAEs will help clinicians to manage these events more effectively and enable assessments of the safety of treatment resumption after irAE resolution.

## Data Availability Statement

The datasets presented in this study can be found in online repositories. The names of the repository/repositories and accession number(s) can be found below: https://www.ncbi.nlm.nih.gov/sra/PRJNA779584.

## Ethics Statement

The studies involving human participants were reviewed and approved by The Ethic Committee of Second Xiangya Hospital at Central South University. The patients/participants provided their written informed consent to participate in this study.

## Author Contributions

All authors made a significant contribution to the work reported, whether that is in the conception, study design, execution, acquisition of data, analysis, and interpretation, or in all these areas; took part in drafting, revising, or critically reviewing the article; gave final approval of the version to be published; have agreed on the journal to which the article has been submitted; and agree to be accountable for all aspects of the work.

## Funding

This work was supported by the National Natural Scientific Foundation of China (82003883). Natural Science Foundation of Hunan Province China (Grant No: 2017JJ3462, 2020JJ5822, 2021JJ40847).

## Conflict of Interest

The authors declare that the research was conducted in the absence of any commercial or financial relationships that could be construed as a potential conflict of interest.

## Publisher’s Note

All claims expressed in this article are solely those of the authors and do not necessarily represent those of their affiliated organizations, or those of the publisher, the editors and the reviewers. Any product that may be evaluated in this article, or claim that may be made by its manufacturer, is not guaranteed or endorsed by the publisher.
