# Supplementary material for: Intestinal Microbiome Associated With Immune-Related Adverse Events for Patients Treated With Anti-PD-1 Inhibitors, a Real-World Study
Source: Front Immunol. 2021 Dec 16;12:756872. doi: 10.3389/fimmu.2021.756872 (PMC8716485; doi:10.3389/fimmu.2021.756872)
Supplement: Supplementary file 1 [file DataSheet_1.docx]

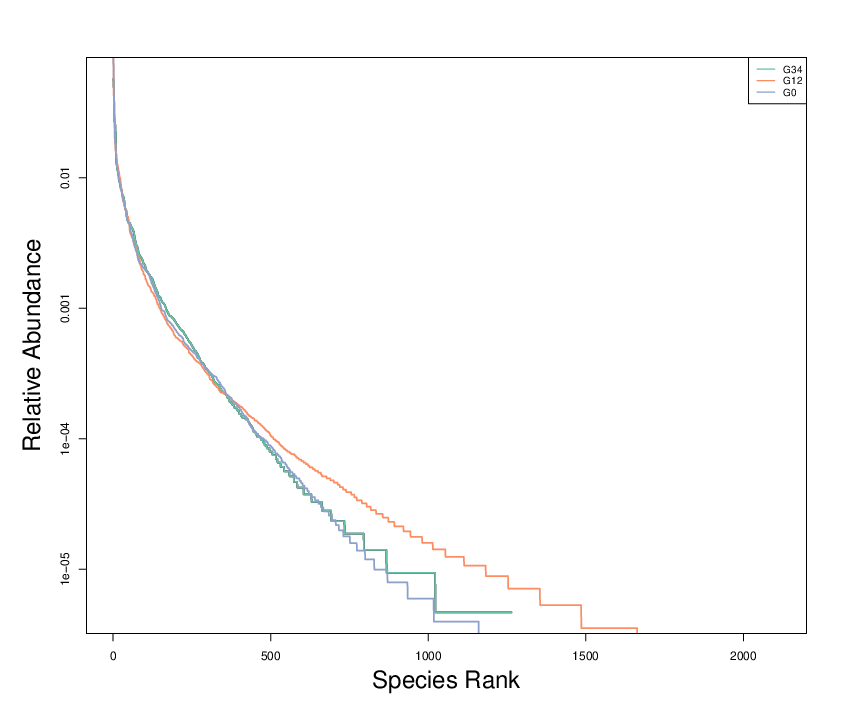


Fig. S1. Coverage of sequencing data among groups. Rare fraction curves of the number of OTUs observed among groups.


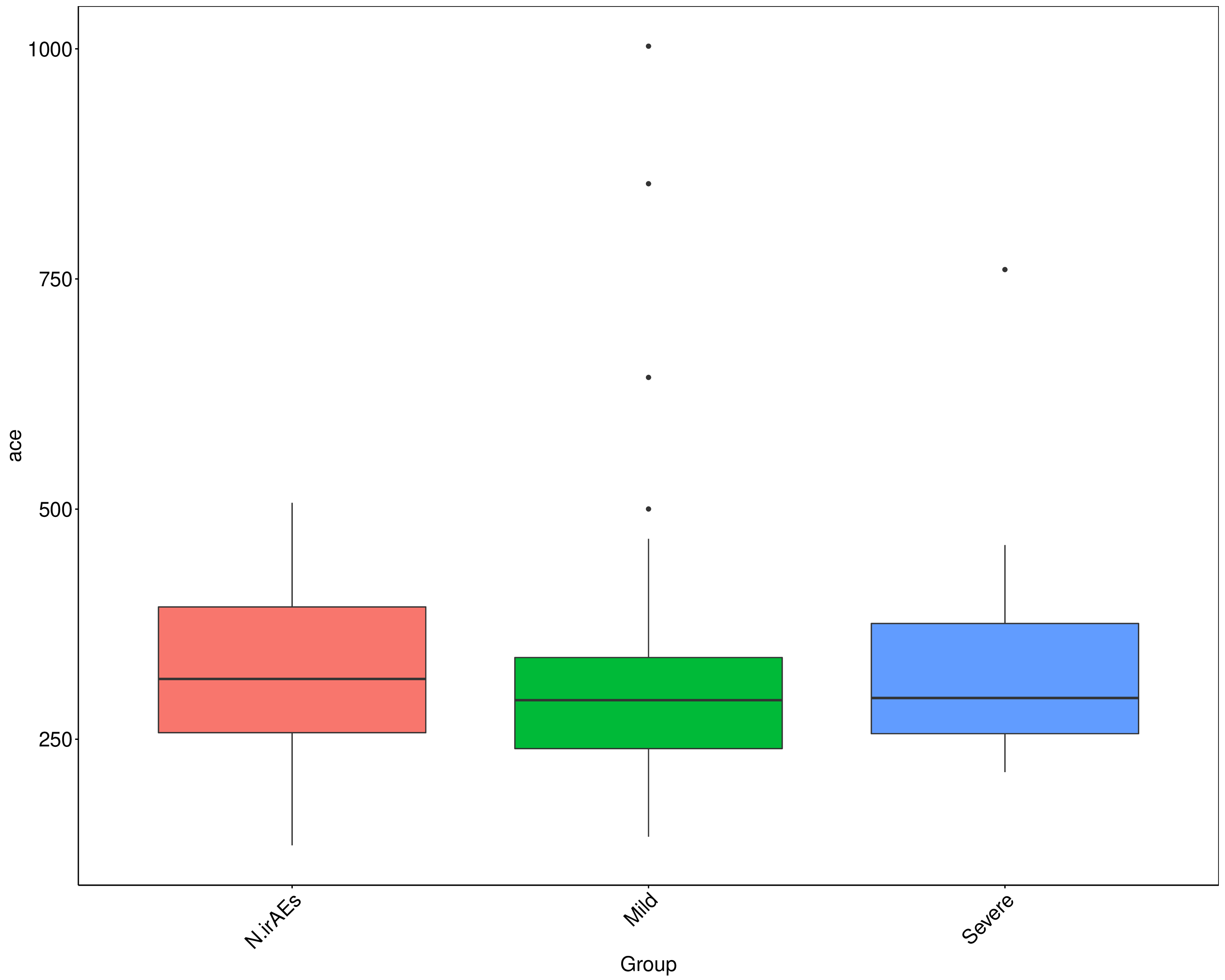

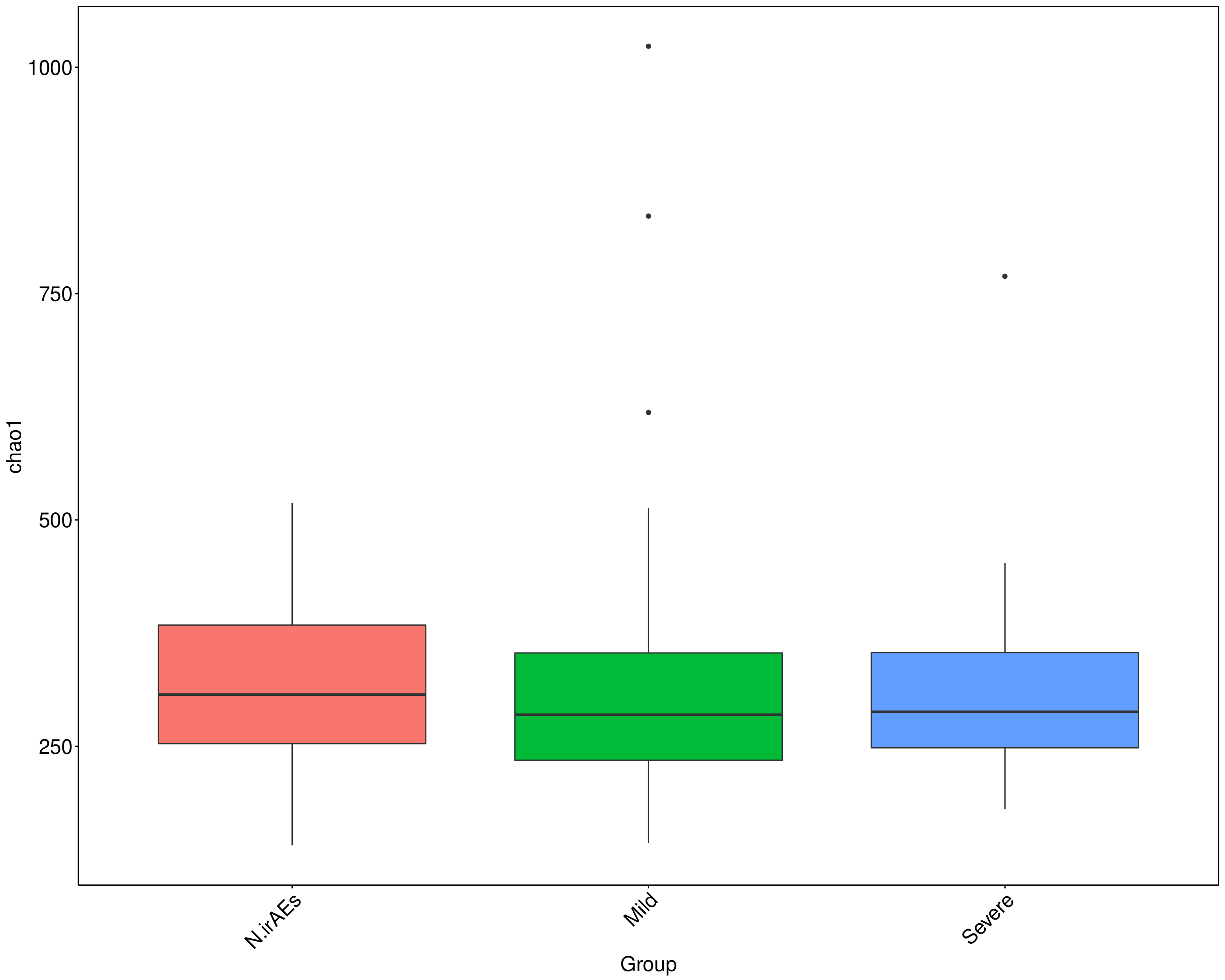


**D**

**C**

**A**

**B**


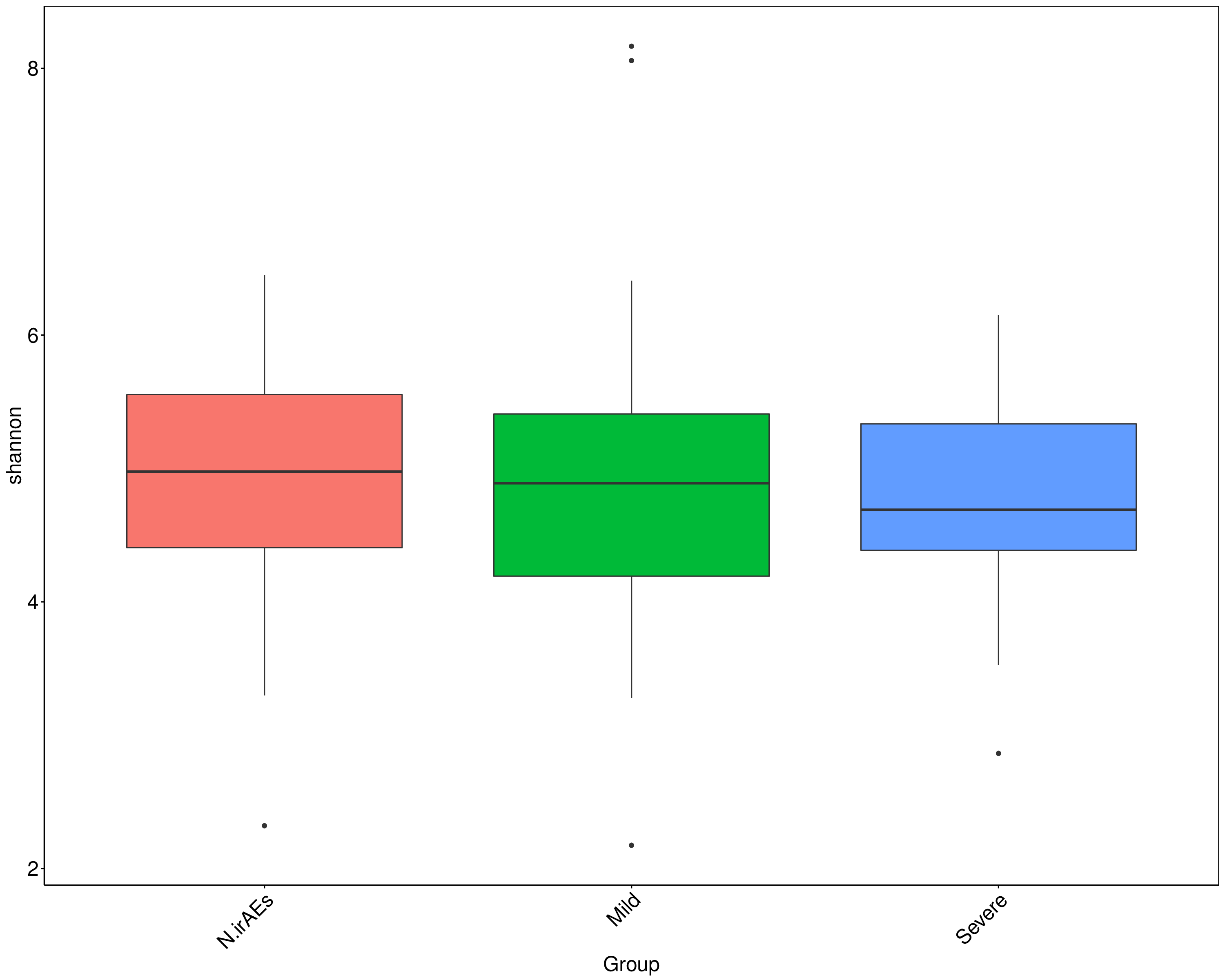

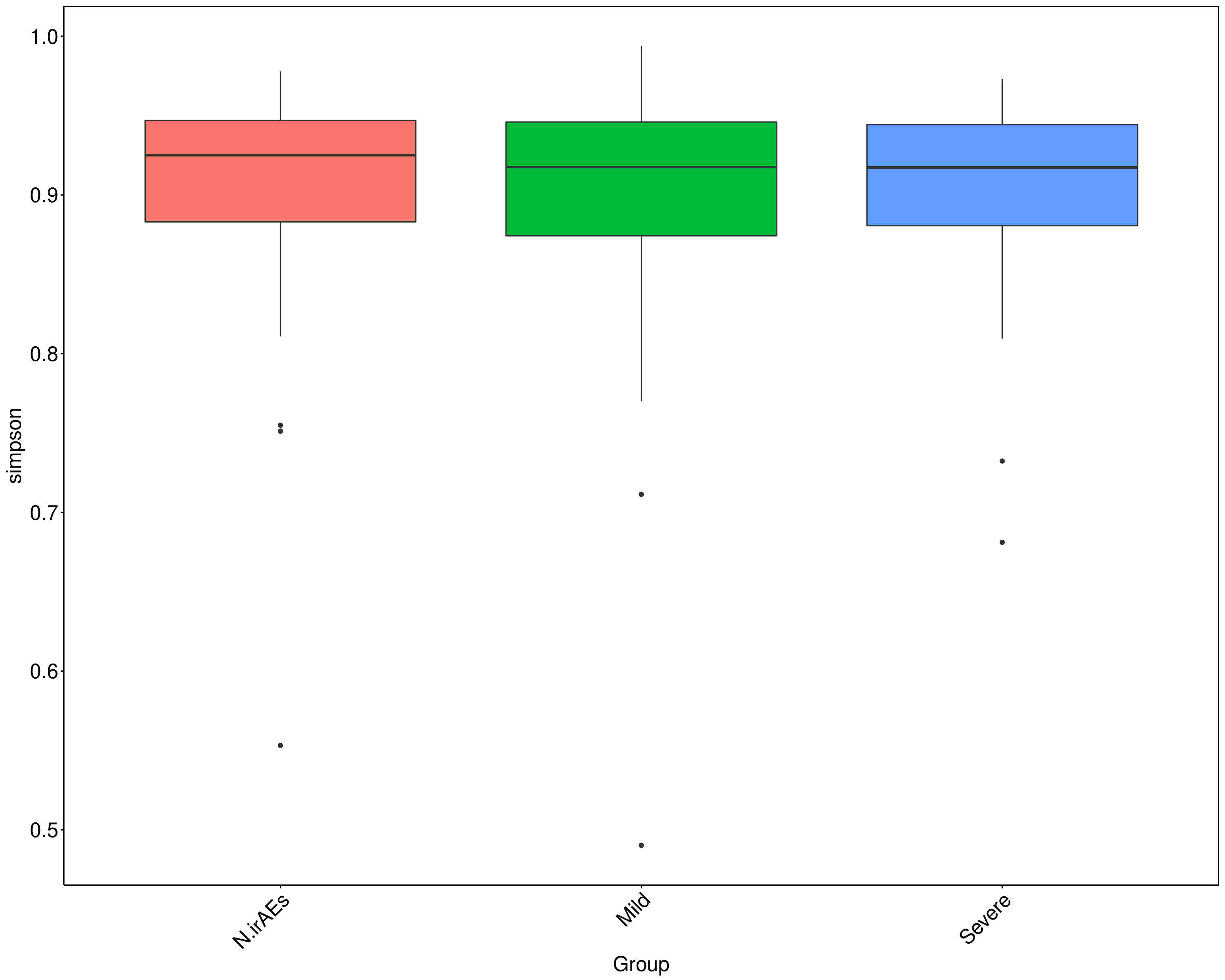


Fig S2. Comparison of α-diversity among three groups. **A**: ACE index **B**: Chao1 index **C**: Simpson index **D**: Shannon index.


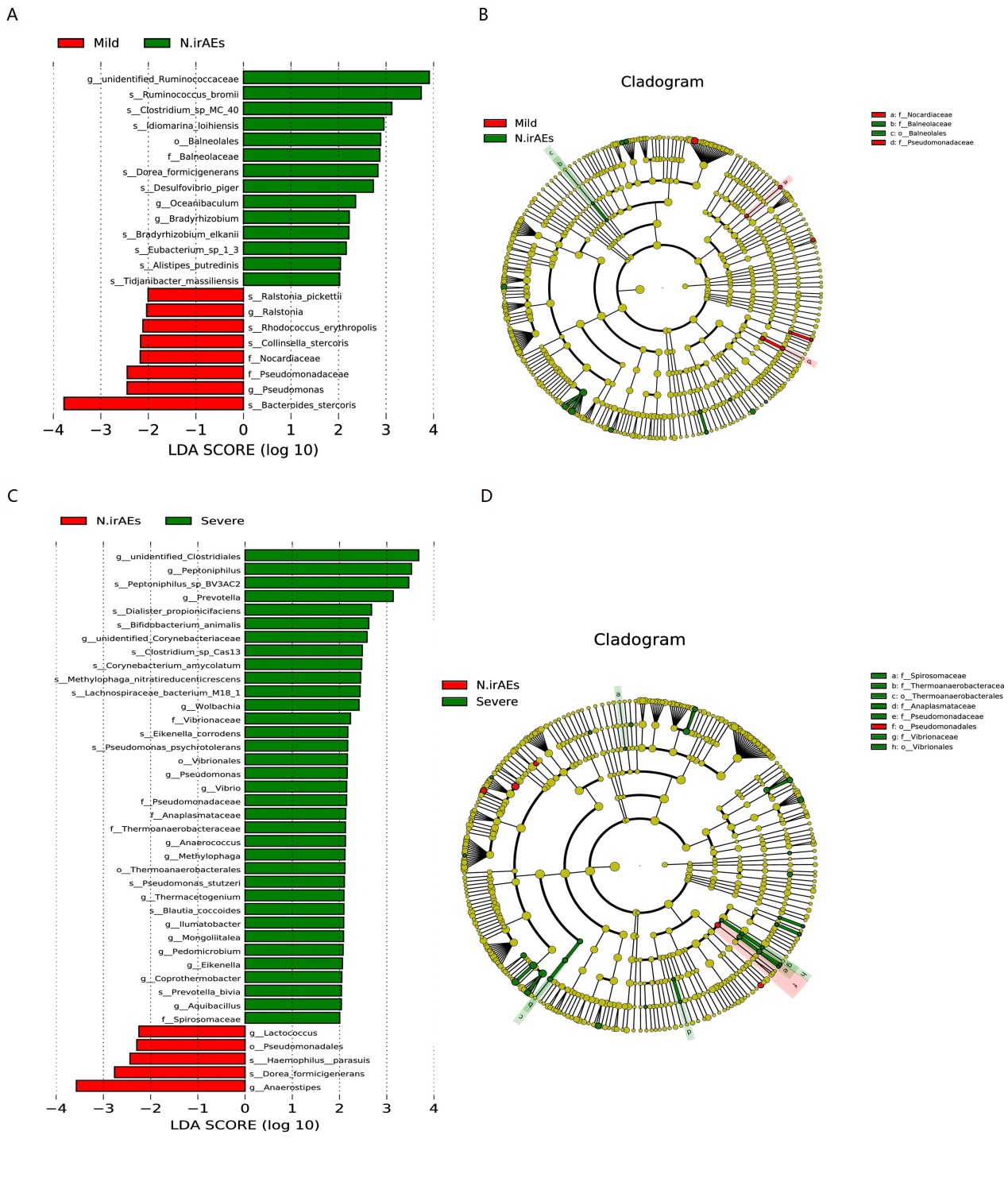


Fig. S3. Bacterial biomarkers of patients without irAEs and those suffered mild or severe irAEs.

**A:** LDA effect size (LEfSe) results represented significantly different in abundance of gut bacteria between the N-irAEs and mild irAEs groups. Significant differences are indicated: Wilcoxon rank sum test. Colour indicates the group in which a differentially abundant taxon is enriched (red: mild irAEs group, green: N-irAEs group). B: LDA effect size (LEfSe) tree results showed the significantly different of bacteria in abundance between N-irAEs and mild irAEs groups and indicated the effect size of each differentially abundance bacterial taxon between groups. C: LDA effect size (LEfSe) results represented significantly different in abundance of gut bacteria between the N-irAEs and mild irAEs groups (red: N-irAEs group, green: severe irAEs group). D: LDA effect size tree results showed the significantly different of bacteria in abundance between N-irAEs and severe irAEs groups.


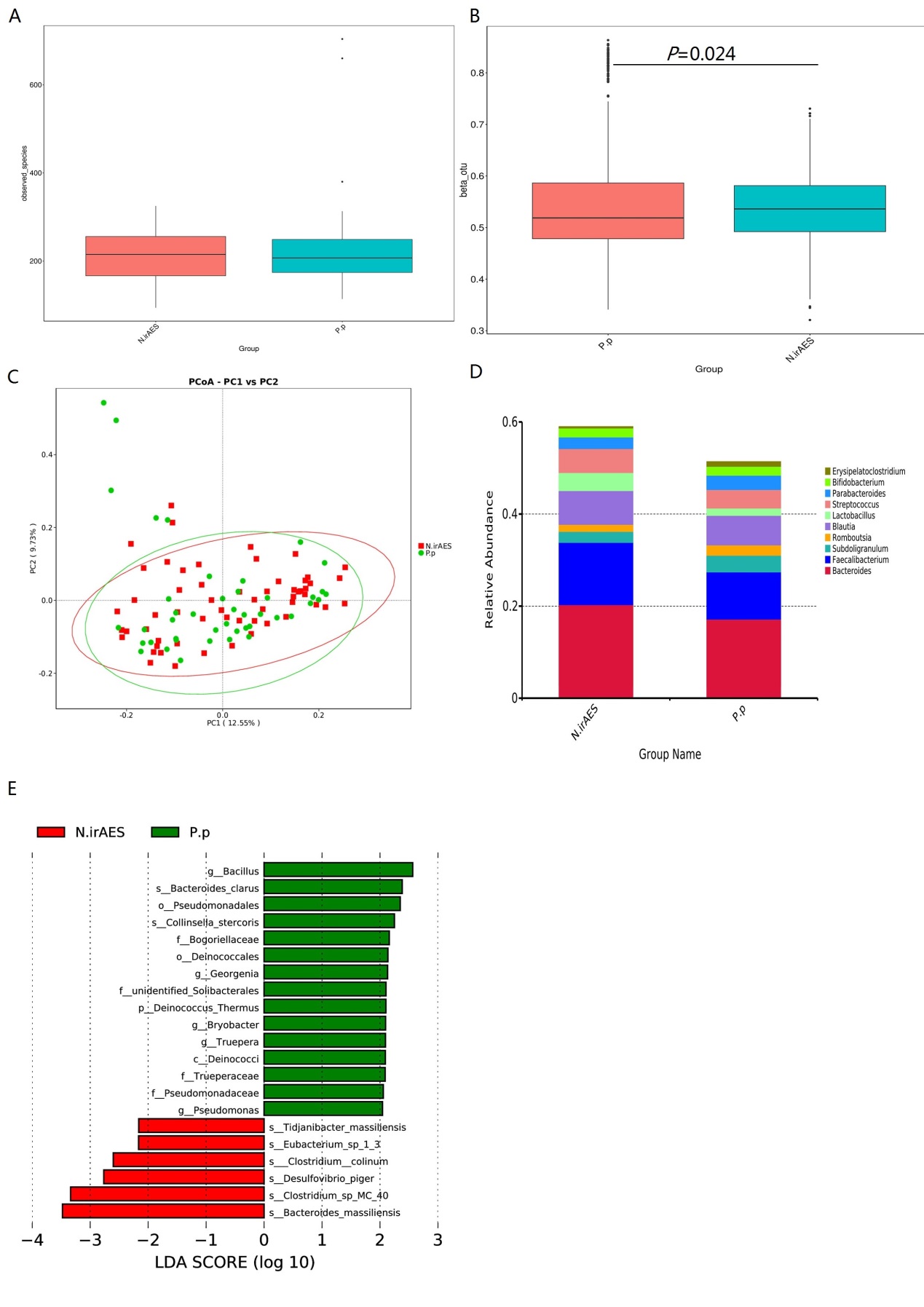


Fig S4. Gut microbiome composition for patients without irAEs and those with pruritus. A. Alpha-diversity for patients from each group via Shannon index. The bold line represents the range from the hinge. B. Beta-diversity for patients of each group by the weighted unifrac analysis. Significant differences are indicated: wilcox rank sum test. Note that all findings for beta-diversity are statistically significant. (C) PCoA test was used to measure the shift in intestinal bacterial composition profile between groups. (D) The relative abundance of the top 10 bacteria at the genus level between groups, ordered by the most abundance taxa across the cohort. (E) LDA scores of bacterial biomarkers between patients without irAEs and these suffered from pruritus toxicities, calculated by using LEFSe.


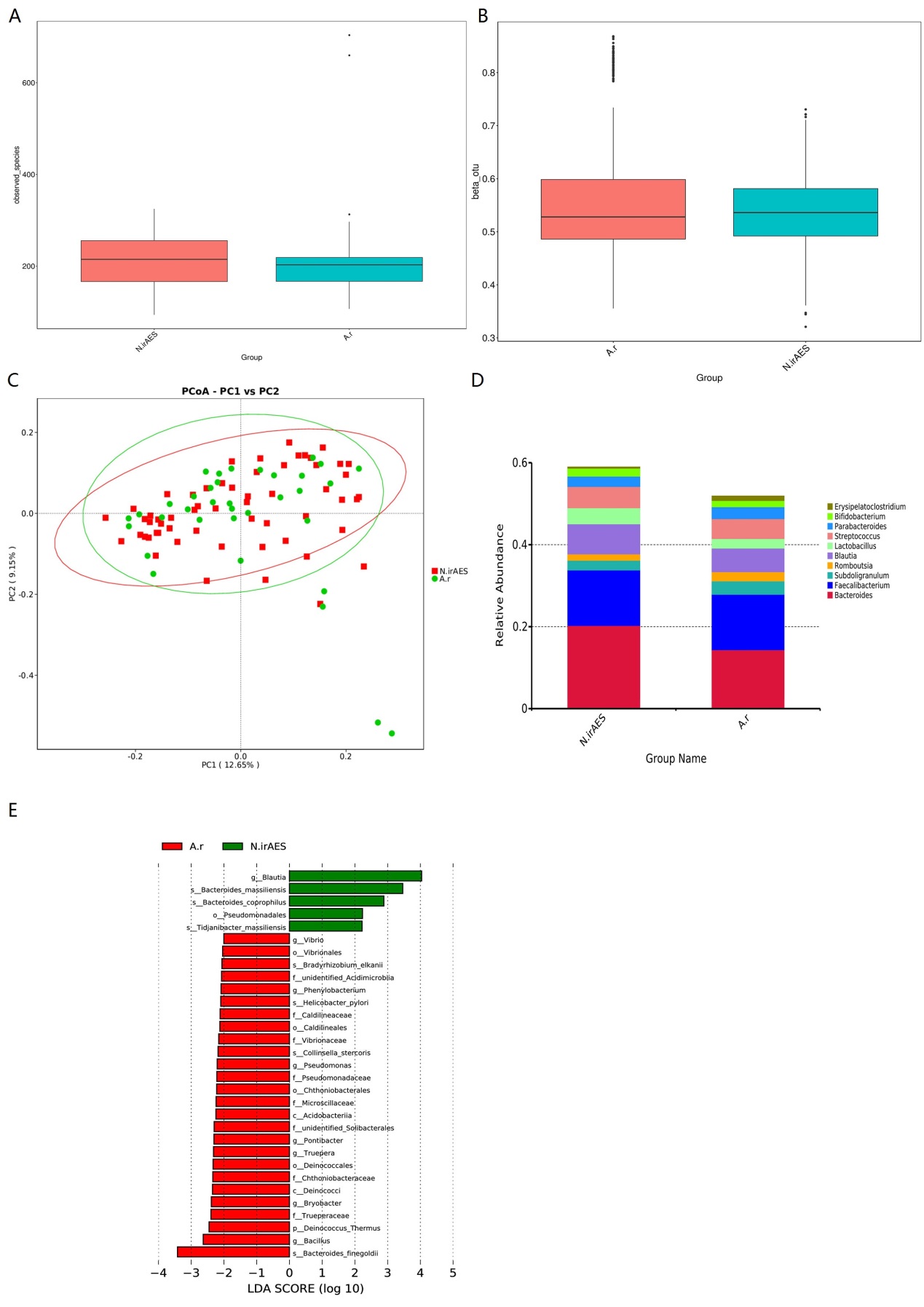


Fig S5. Gut microbiome composition for patients without irAEs and those with rash. A. Alpha-diversity for patients from each group via Shannon index. The bold line represents the range from the hinge. B. Beta-diversity for patients of each group by the weighted unifrac analysis. Significant differences are indicated: wilcox rank sum test. Note that all findings for beta-diversity are statistically significant. (C) PCoA test was used to measure the shift in intestinal bacterial composition profile between groups. (D) The relative abundance of the top 10 bacteria at the genus level between groups, ordered by the most abundance taxa across the cohort. (E) LDA scores of bacterial biomarkers between patients without irAEs and these suffered from pruritus toxicities, calculated by using LEFSe.


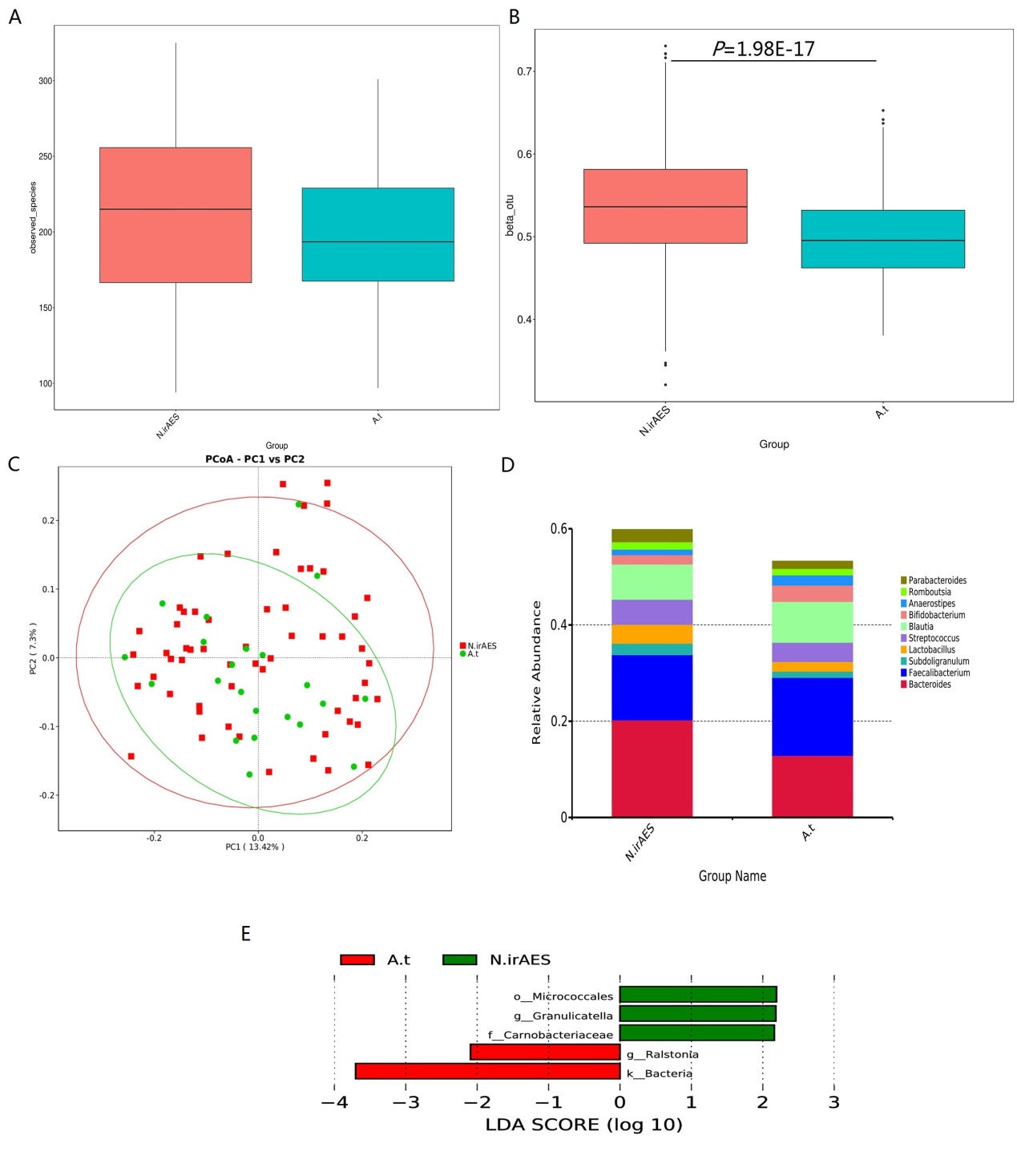


Fig S6. Gut microbiome composition for patients without irAEs and those with thyroid dysfunction. A. Alpha-diversity for patients from each group via Shannon index. The bold line represents the range from the hinge. B. Beta-diversity for patients of each group by the weighted unifrac analysis. Significant differences are indicated: wilcox rank sum test. Note that all findings for beta-diversity are statistically significant. (C) PCoA test was used to measure the shift in intestinal bacterial composition profile between groups. (D) The relative abundance of the top 10 bacteria at the genus level between groups, ordered by the most abundance taxa across the cohort. (E) LDA scores of bacterial biomarkers between patients without irAEs and these suffered from pruritus toxicities, calculated by using LEFSe.


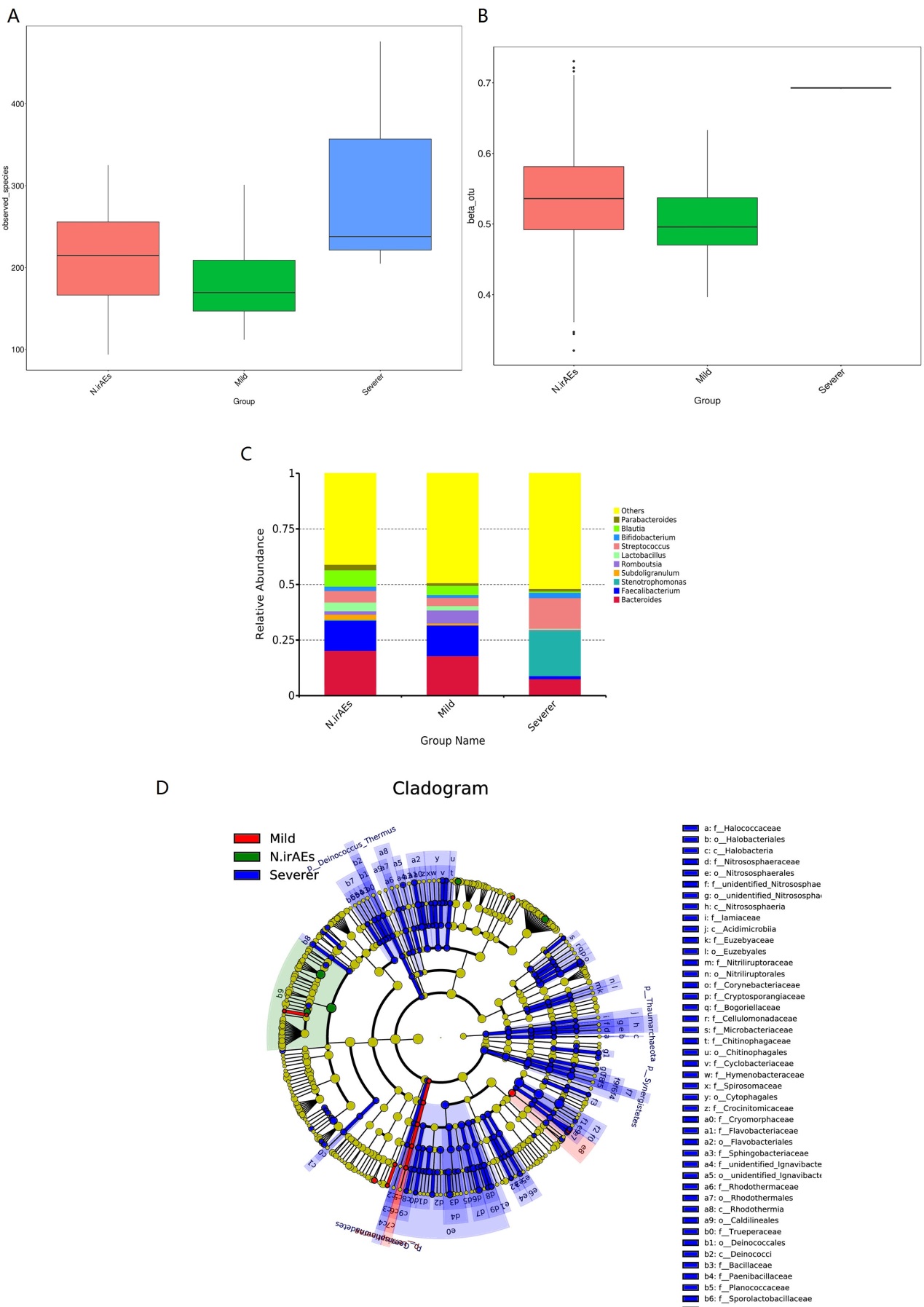


Fig S7. Gut microbiome composition for patients without irAEs and those with mild or severe diarrhea. A. Alpha-diversity for patients from each group via Shannon index. The bold line represents the range from the hinge. B. Beta-diversity for patients of each group by the weighted unifrac analysis. Significant differences are indicated: wilcox rank sum test. Note that all findings for beta-diversity are statistically significant. (C) The relative abundance of the top 10 bacteria at the genus level between groups, ordered by the most abundance taxa across the cohort. (D) LDA scores of bacterial biomarkers between patients without irAEs and these suffered from mild or severe diarrhea, calculated by using LEFSe.


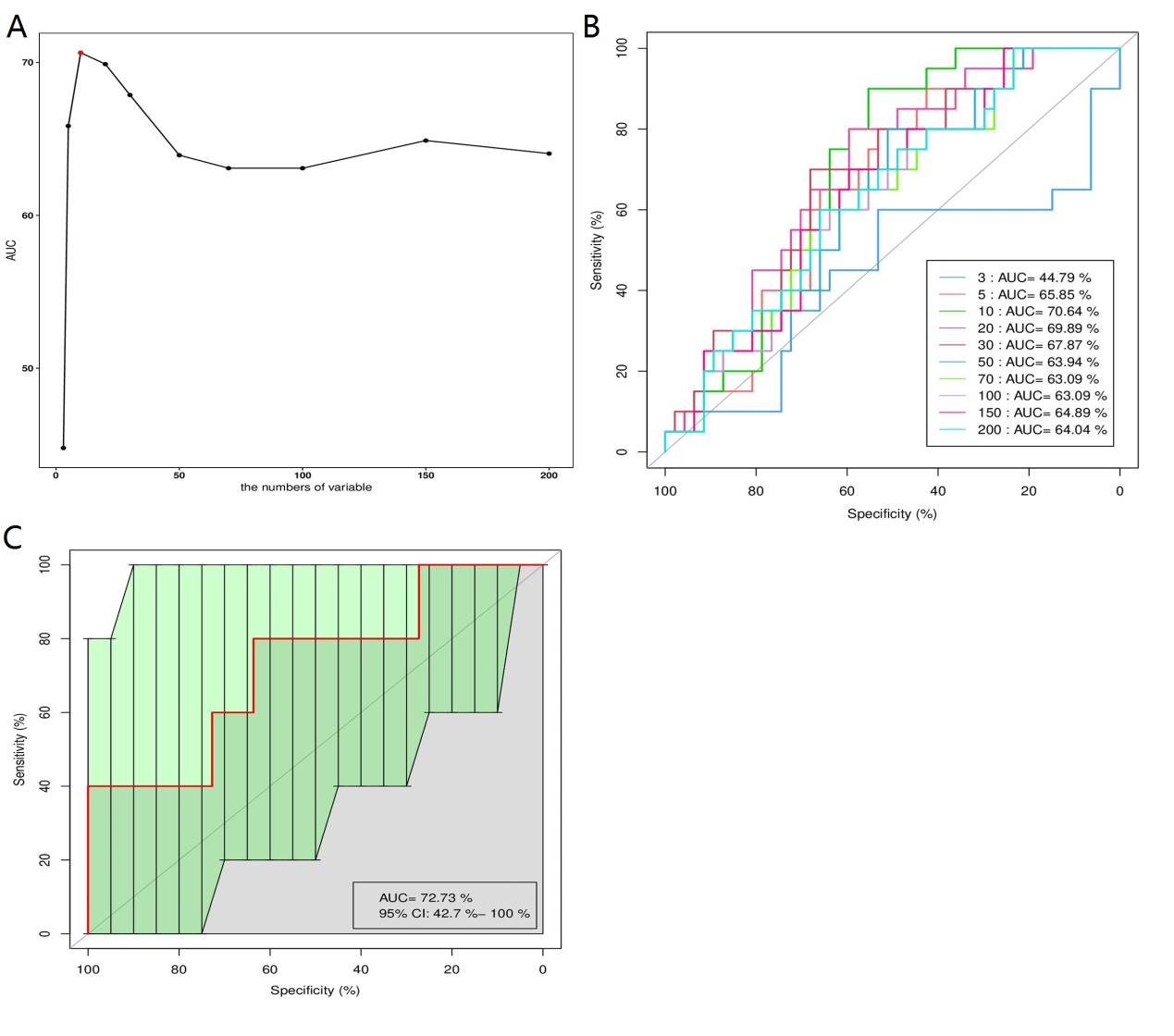


Fig. 8S. Classification model for severe irAEs based on gut microbes. **A:** The AUC value of different numbers of bacterial features. **B:** The ROC curve of support vector machine (SVM) algorithm classification models using different numbers of gut bacteria.


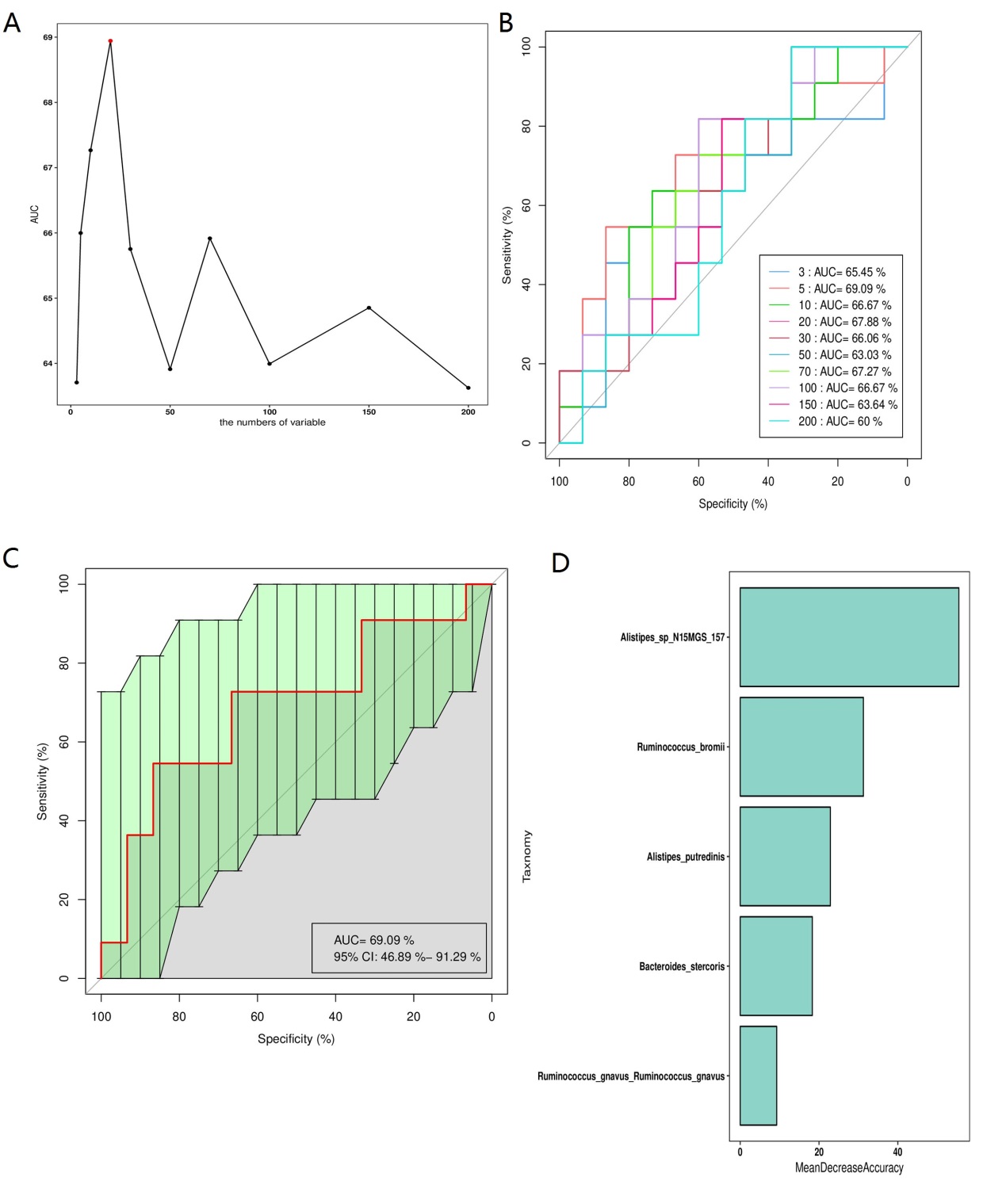


Fig. 9S. Classification model for patients without irAEs or with mild irAEs based on intestinal microbes. **A:** The AUC value of different numbers of bacterial features. **B:** The ROC curve of SVM algorithm classification models using different numbers of gut bacteria. C: The ROC curve of SVM classification model using the species abundance. D: The mean decrease accuracy of each enrolled bacteria taxa in the SVM classification model.
